# The ‘DPIA+’: Aligning data protection with UK equality law

**DOI:** 10.1016/j.clsr.2025.106212

**Published:** 2025-11

**Authors:** Miranda Mourby

**Affiliations:** a University of Oxford, Faculty of Law, St Cross Building, Oxford, OX1 3UL, UK; b University of Sheffield, School of Law, Bartolomé House, Winter Street, S3 7ND, UK

**Keywords:** Data protection, Data protection impact assessment, Discrimination, Equality law

## Abstract

In recent years, data protection scholarship has moved beyond the assumption that the General Data Protection Regulation (‘GDPR’) is solely concerned with individual rights. Tools such as the Human Rights Impact Assessment (‘HRIA’) and the Fundamental Rights Impact Assessment ('FRIA') have been promoted to apply the GDPR more expansively, capturing broader societal harms that may flow from personal data processing. These tools can widen the scope of the GDPR’s Data Protection Impact Assessment (‘DPIA’) through aligned consideration with human rights law. They have been outlined at an international level, but require adaptation to national contexts in practice.

This article advances the discussion in three ways. First, it develops a jurisdiction-anchored expansion of the DPIA (‘DPIA+’) by integrating the UK Public Sector Equality Duty in s.149 Equality Act 2010. Second, it highlights equality law as both overlapping with, and distinct from, human rights law. In the UK, equality law imports a proactive duty to investigate risks of discrimination, while also providing an evaluative template in the form of an Equality Impact Assessment. Finally, it considers the distinctive value of an equality-inflected DPIA+ in life-and-death contexts, such as the Covid-19 pandemic.

The open-ended term ‘DPIA+’ acknowledges that various legal frameworks may supplement a DPIA in each national context. The central argument, however, is that equality and human rights law should be considered together when augmenting a DPIA, as both can help identify and address risks of discrimination in personal data processing.

## 1 Introduction

The last decade has seen broad, multidisciplinary discussion of ‘Big Data’: the algorithmic analysis of large volumes of information about people, with the promise of yielding faster (even, perhaps, better) insights.^1^ An argument often made within this discourse is that privacy and data protection instruments are too ‘small’, too granular,^2^ to regulate Big Data. Broadly speaking, these laws are often seen as typified by data subjects’ rights, and thus centred around the interests and choices of the individuals identified by the data. As such, the general argument goes, data protection law atomises regulation into consideration of the risks and harms to each individual, who may or may not have the information, insight and expertise to regulate the use of their own data.^3^ A common corresponding claim is that data protection therefore provides inadequate protection for the interests of groups,^4^ for individuals subject to downstream consequences of data processing,^5^ or for society as a whole. A range of supplements/alternatives have been proposed to remedy this alleged deficiency, from additional data subject rights to help individuals control algorithmic inferences about them,^6^ to algorithmic impact assessments,^7^ and governance models such as data trusts^8^ or systemic oversight.^9^

In an alternate line of scholarship, some authors have argued that existing data protection law can be interpreted more expansively in its application, to capture the wider social consequences of data processing. By focusing instead on the evaluative obligations under the General Data Protection Regulation^10^ (‘GDPR’), they do not assume that data protection is solely concerned with individual rights. In an expansion of the GDPR’s risk-assessment obligations, Mantelero,^11^ as well as Ortalda and De Hert,^12^ have advocated for a ‘Human Rights Impact Assessment’ evaluation tool. This ‘HRIA’ would provide a voluntary human rights framework to bear on the GDPR’s Data Protection Impact Assessments (DPIAs),^13^ The HRIA tool is outlined as a high-level baseline,^14^ which can be adapted to local contexts. When adapted to local laws, the HRIA should permit DPIAs to capture not only the immediate quality and security of processing,^15^ but also any foreseeable downstream consequences. Similarly, Bertaina and colleagues view the forthcoming Fundamental Rights Assessment under the EU’s AI Act as a promising supplement to a DPIA, particularly because it highlights the impact of processing on vulnerable groups.^16^ Naturally, the AI Act will only apply within the EU, making this model a useful precedent, but also a voluntary mechanism for non-EU jurisdictions like the United Kingdom.

An emerging consensus seems to suggest that the GDPR’s baseline requirements of a DPIA require some kind of supplement to permit consideration of the longer-term risks of data processing.^17^ In support of this line of scholarship, this article proposes equality law as an additional supplement to the DPIA, and illustrates its values with references to the particular context of healthcare in the UK. In the UK public sector, failure to consider group impacts is not only a DPIA concern but also a potential breach of obligations under the Equality Act 2010. Equality legislation can help provide a concrete template to apply fundamental rights within a DPIA. It imports comprehensive guidance on risks of discrimination,^18^ but also an evaluative template in the form of the ‘Equality Impact Assessment’.^19^ The final form of impact assessment discussed here, an ‘EIA’ is not mandatory under the Equality Act 2010. However, it is a common way for public authorities to evidence and ensure compliance with a legal obligation that *is* mandatory: the public sector equality duty under s.149 Equality Act 2010. Within the UK’s National Health Service (‘NHS’), it has been adapted into an ‘Equality and Health Inequalities Impact Assessment,’ to further include the health-specific equality requirements of the Health and Social Care Act 2012.^20^

Amidst this dense thicket of potential forms of impact assessment, this article is open-ended as to the range of national legislation that could supplement a DPIA in each local context. Hence the expansive term ‘DPIA+.’ Here, the Equality Act 2010 is taken as a national example of an equality framework, which provides an important (and, thus far, under-examined) dimension of the human rights considerations that can expand a DPIA. The UK’s ‘EIA’ essentially adopts core structure I advocate in my broad ‘DPIA+’ approach: breaking down potential risks of discrimination or exclusion according to groups with particular protected characteristics (e.g. in terms of age, race or disability), and providing a systematic account of how these risks will be mitigated.^21^

The process of adapting an HRIA-type model to local contexts is undeniably complex— especially given the multiplicity of laws which could be brought to bear on an expanded DPIA. However, I have elected to consider the specific application of UK equality law to a DPIA in the context of Covid-19. This is because of a) the serious impact of any flaws in life-and-death decision-making such as emergency resource allocation, as well as b) the ready model of the ‘EIA’ which could be brought to bear on these risks.

The next section explains in more detail why the risk of bias in public-sector data warrants the enhanced safeguard of (some form of) a DPIA+.

## 2 Big data in the public sector

Although ‘Big Data’ is, at times, used in a precise socio-technical sense within academic and policy literature, the central concern of this paper is broader. More generally, I refer to ‘Big Data’ in the public sector as covering the use of large datasets by public authorities Rather than referring to a specific processing technology, I agree with Mantelero’s use of the term to denote the expansive scope of data-driven models employed in decision-making, and their implications for fundamental rights.^22^ In the present analysis, the key right at stake is the right not to be subjected to discrimination.^23^

### 2.1 The risks of public-sector automation

The UK is taken as the case study for this paper, to show the value of an expanded DPIA within a particular national framework. However, the risks of rolling out software to evaluate people, and allocate public benefits, are not confined to the UK. A vivid example comes from the Dutch welfare system, which introduced algorithms that inaccurately labelled dual national and/or ‘foreign-sounding’ recipients as likely benefit frauds. The fault in question can be characterised as one of bias, as the software used ethnicity as an (illegal) proxy for dishonesty when making its assessments.^24^ As with the UK’s own Post Office scandal,^25^ the victims of these automated errors reported lives ruined, and even suicide.^26^ It is entirely possible for comparable bias to occur in other public authorities: the UK’s Department for Work and Pensions has also reportedly used an AI to detect benefit fraud, and subsequently undertaken a fairness analysis that showed up bias on the basis of age, disability, marital status and nationality.^27^

There are, undoubtedly, advantages to automated analysis within public services— for example, by detecting disease early or improving patient screening programmes.^28^ But just as it is true to say that ‘data saves lives,’^29^ inaccurate and discriminatory use of data can evidently ruin them, and even cut them short. There is no evidence that the algorithms used by UK public authorities so far^30^ have caused harm to an equivalent degree as the Dutch welfare automation. But, clearly, some life-and-death processes are entrusted to automated systems. As an example, the Information Commissioner’s Office (the data protection regulator for the UK) noted that an inadequately-tested code was released into the NHS transplant system and failed to match five patients before it was caught.^31^ Again, fortunately, these five patients were reported not to have suffered any harm, but it is reasonable to infer that any flaws in software applied nationally could have had much wider consequences.

### 2.2 Automation & Covid-19

The case study explored in this paper is the UK’s NHS Covid-19 Data Store. This resource is not explored to make any suggestion of discrimination in the NHS response to Covid-19. Rather, the risks of under-representation of some demographics that have since become apparent are highlighted, to establish a prima facie case for a risk of bias, worthy of evaluation to prevent discrimination.

The Covid-19 Data Store was urgently set up to help the UK government decide how to distribute resources at the beginning of the pandemic.^32^ We know, now, that some groups fared worse in the pandemic, and had disproportionately high rates of mortality.^33^ There was clearly a risk—and to be clear, I only propose it as a *risk*—that bias in the data could have misrepresented the prevalence of Covid-19 in some populations, and thus misrepresent their need for (for example) more clinical staff in their area, or prioritisation for vaccination.^34^ This risk has been explored, retrospectively, in epidemiological studies. But the crucial point for this paper is that the GDPR requires that accuracy, fairness and risks of discrimination be considered *prospectively*, particularly within a DPIA. A DPIA was conducted by NHS England for the Covid-19 Data Store, but the published document does not evidence any reflection on risks associated with bias in the data.^35^ We cannot assume, from this, that no such reflection took place. However, under the DPIA+ model I advocate, any consideration of discrimination risks would be documented more explicitly, alongside any immediate data security concerns. This is the standard set by the precedent of an ‘Equality Impact Assessment,’ or the NHS’ modified ‘Equality and Health Inequalities Impact Assessment,’ which are often published with this level of transparent, structured reflection on the impact on particular groups.^36^

The UK government announced in March 2020 that it would create an NHS Covid-19 ‘Data Store’ from information routinely collected as part of the health service. This ‘Store’ would use data from the NHS (and other public authorities) to create ‘dashboards’ with predictions developed through artificial intelligence. These dashboards would in turn provide a ‘*single source of truth*’^37^ about the spread of the coronavirus in England. Decisions made on the basis of these dashboards would be significant, even (it was suggested) to the point of diverting patients and critical resources between hospitals based on their predictions. The DPIA prepared by NHS England makes it clear that the Data Store was constructed using fully identifiable personal data, although access was restricted to a small number of staff for pre-processing and validation before the data were anonymised or pseudonymised.^38^ While it is not entirely clear to what extent the data in the Store were anonymised (and thus no longer personal data) or pseudonymised (and therefore still within the scope of the GDPR), this distinction is not essential for the purposes of this article. What matters for the present analysis is the ‘input’ stage of processing, in which patients’ personal data were used to generate the dashboards, allowing any limitations in the underlying data to be fully evaluated.

The Data Store was ‘Big Data’ in the classic sense of aggregating a variety of data to build a bigger picture of a national trend, which would be updated daily with fresh information.^39^ Machine-learning tools were built into the platform using Microsoft Azure to make predictions about the spread of the virus. By going beyond a factual picture of the outbreak, and attempting to anticipate it using AI, the government was, in essence, conducting fast-paced, real-time epidemiology on the spread of Covid-19. The tone of the announcement of the store conveys the urgency of the undertaking, and the vital resources at stake:

*The NHS is facing an unprecedented challenge. Responding to the Covid-19 crisis will require everything we have and more. In the fight against this pandemic, decision-makers will need accurate real-time information. To understand and anticipate demand on health and care services, we need a robust operating picture of the virus, how it’s spreading,****where it might spread next****and how that will affect the NHS and social care services. On the supply side, we need to know****where the system is likely to face strain****first, be that on ventilators, beds or staff sickness* (emphasis added)*.*^40^

The key point, here, is that the predictions made within the Store could have implications for the distribution of resources. The risk arises from the phenomenon of ‘Big Data’ in the broader sense outlined above. We now know that different patient groups experienced different outcomes in the first wave of Covid-19. While it is not the aim of this article to raise any causative links between these outcomes and the allocation of resources, these differences are explored through a lens of risk. This is to make a case that the risk of disparity could have been anticipated, and usefully explored in a DPIA (as enhanced by equality law). To explain why, however, the next section provides more information on the nature and purpose of the DPIA.

## 3 The data protection impact assessment

The Data Protection Impact Assessment was introduced by the EU’s General Data Protection Regulation in 2018. As such, it is a relatively new instrument, albeit it was preceded by a (non-mandatory) Privacy Impact Assessment under the previous Data Protection Directive.

It is not misleading to describe the DPIA as an ‘obligation,’ as the ‘Big Data’ scenarios of large-scale processing discussed in this paper will trigger the DPIA as a mandatory requirement under Article 35 GDPR. A data controller must undertake a DPIA before they embark on large-scale processing of ‘special category’^41^ data. If a high risk to data subjects is identified, the controller can only proceed subject to the instructions of the national data protection regulator.^42^ The DPIA process is clearly intended by the drafters of the GDPR to be a serious gatekeeping exercise for high-risk data processing, with much sharper ‘teeth’ than mere good-practice guidance. The mandatory nature of these legal requirements is a key to their value as safeguards— providing a reassuring contrast to the voluntary measures for developers the UK Government has otherwise proposed in the context of the development of AI.^43^

### 3.1 DPIAs and ‘Downstream’ consequences

This paper concurs with other authors^44^ that the DPIA can be understood expansively, as concerned with the wider consequences of data processing, and not just its immediate quality and security. To accept this position, it is necessary to understand the GDPR as an instrument which is not entirely focused on individuals, and can also regulate equal treatment between groups. While individual data subjects are important, a large proportion of the GDPR is devoted to the obligations of the organisations which control data: ‘data controllers.’^45^ The rights of individuals identified by the information—‘data subjects’— in fact make up only 9 of its total 99 Articles.^46^ Delacroix and Lawrence suggest the resulting data controller-data subject relationship is ‘feudal,’^47^ but this balance of provisions does not need to be understood in this way. As these authors themselves acknowledge,^48^ the individual cannot be fairly or reasonably expected to regulate all of the data processing of which their information may form a part.

Rather, it is the data controller—not any of the individuals identified by the information—who has the necessary overview of all the information they process, the time and resources to evaluate associated risks to individuals and groups, and most importantly, the legal responsibility to ensure their lawful use of information. Individuals have some rights to modify how information about them—and only them—is used, as a recognition of their personal right to privacy and data protection.^49^ But, as the UK Supreme Court has pointed out, this does not mean that data protection law as a whole is predicated on the idea of individual control.^50^

Ultimately, data controllers have the responsibility to ensure that their use of personal information is lawful. They must make this determination with reference to the GDPR’s broad principles of transparency, lawfulness, data minimisation, accountability, and (crucially for the anti-discrimination questions raised in this paper) fairness.^51^ These principles, I suggest, go beyond the interests of the individual and address the more widespread implications of data processing.

As a key example, data protection impact assessments are not limited to, or even primarily focused on, the risks to a collection of individuals. As the UK’s Information Commissioner’s Office (‘ICO’) notes in their guidance:

*It is also important that you don’t just consider obvious and immediate tangible damage to people. But also more subtle intangible harms and how the system might affect people's rights and freedoms more generally. This includes any impact on society as a whole. For example, DPIAs require you to consider risks to rights and freedoms of all those that the system might affect.*^52^

This guidance provides an important gloss on the text of the GDPR. By opening up the scope of consideration beyond the rights of the individuals identified by the information processed, the ICO is also making an important point about the broader purpose of data protection law. Data subjects are clearly important, as the people most immediately impacted when information about them is used. But they are not the only people impacted by the potential downstream consequences of—for example—a policy decision made using some people’s personal data, but then applied much more widely. Hence why McMahon and colleagues advocate for new bodies to deal with the downstream consequences of Big Data.^53^

I am not dismissing the question of further regulation for these kinds of downstream consequences. My argument, however, is that existing data protection legislation— and, in particular, the DPIA— contains untapped potential to address consequences of processing which go beyond the individuals identified by the information. The ICO’s guidance provides a helpful nudge to data controllers to consider the ‘bigger picture’ of their processing—such as the risk of data bias skewing their downstream policy and decision-making. This nudge deserves highlighting, amplification, and (crucially), formalisation, so that these wider considerations are considered systematically. The next subsection explains further why the DPIA is a helpful nexus for this formalisation.

### 3.2 DPIAs and fairness

As a GDPR obligation, a DPIA must be conducted in a way that seeks alignment with the principles of the regulation. Of the overarching principles set out in Article 5 GDPR, the fairness principle^54^ is particularly important for our current purposes. It is a principle which some place at the heart of data protection law, as a distinct body of doctrine. For example, in seeking to define the essential core of the right to data protection, as distinct from privacy, Tzanou lands on ‘fair processing’ as a starting point, and also includes the principle of non-discrimination.^55^ Dove broadly concurs with this characterisation of data protection, as distinct from privacy.^56^ Lynskey, in considering the ‘added-value’ of the right to data protection, suggests that there are some harms that data protection wards against more effectively, such as the risk of discrimination through proxies or presumptions.^57^

I agree with these authors that the distinction between privacy and data protection is complex, and difficult to pin down, but that there is still a non-symbolic difference between the two.^58^ A final pronouncement on this question is beyond the scope of this paper, but one key aspect of the distinct character of data protection law lies in its attempt to achieve fairness between different actors and stakeholders. Data subjects are given individual rights,^59^ data controllers a carefully codified framework through which to process data lawfully,^60^ and even national regulators have their roles and powers set out.^61^ As the scope of the GDPR goes well beyond the ‘micro’ dynamics of an individual controller and subject, it is less surprising that its core principles include more systematic considerations, such as fairness and accountability.^62^

The GDPR’s conception of fairness seems to be, at the very least, congruent with that in equality law, and it seems from the text of the GDPR that the two overlap. Within the recitals, it is explained that ‘special categories’ of information (the large scale use of which prompts a DPIA^63^) because their use gives rise to a higher risk of discrimination.^64^ Recital 71 of the GDPR contains a long, heavily caveated sentence which truncates to:

*In order to ensure fair and transparent processing in respect of the data subject,* […] *the controller should* […] *secure personal data in a manner* […] *that prevents, inter alia, discriminatory effects*

Additionally, Recital 75 GDPR makes it clear that the risk of discrimination is intended to be considered among the harms the controller should consider as part of a DPIA:

*The risk to the rights and freedoms of natural persons, of varying likelihood and severity, may result from personal data processing which could lead to physical, material or non-material damage, in particular: where the processing may give rise to discrimination*

Binns has characterised the DPIA as a form of ‘meta-regulation’: an attempt by the state to shape corporations’ internal efforts to assess and self-regulate their use of data.^65^ This may well be a useful characterisation of the DPIA in its private-sector manifestation, but does not capture all the dimensions of a public-sector DPIA. Similar to a private company, a public authority will bear its own legal responsibility to comply with their responsibilities as a data controller, and is encouraged to take ICO guidance into account.^66^ However, public authorities will have an additional level of scrutiny in the shape of judicial review of their decisions via the High Court in England and Wales where— as emphasised in this paper— other public law considerations can be brought to bear. It is perfectly possible, for example, for a Claimant to plead a breach of the GDPR and the PSED in relation to the same planning process around a new policy.

The significance of the DPIA should not come as a surprise to anyone with a familiarity with data protection as it has been applied in the UK public sector. It is a record of review of the legality of data processing, which can be considered by a court in the event of judicial review into a new policy.^67^ Failure to conduct an adequate DPIA is a breach of the GDPR, rendering processing unlawful even if it is based on government policy.^68^ The importance of the DPIA when new public policy involves large-scale processing of personal data should not be underestimated, as a superficial, unreflective ‘tick-box’ review of risks to data subjects is unlikely to pass muster within a judicial review. This is illustrated further in the next section.

## 4 Equality law and the DPIA+

The contribution of this paper lies in exploring the added value of equality law as a supplement to a Data Protection Impact Assessment (DPIA), as an overlapping but distinct dimension of fundamental rights. While the legal right to equality in Europe can be traced to the prohibition on discrimination (at least in relation to Convention rights) in Article 14 ECHR, Article 14 cannot be taken as a proxy for European equality law as a whole. As the Council of Europe and the EU Agency for Fundamental Rights have jointly emphasised in their *Handbook on European Non-Discrimination Law*,^69^ EU equality law has followed a separate trajectory, developing through distinct legislative instruments since the 2000s. Although the Court of Justice of the European Union has been influenced by the ECHR since it began to articulate equality principles in the 1960s, significant differences remain in scope, motivation, and historical development between the non-discrimination guarantees in EU law and those in the ECHR.^70^

Although it is possible that EU equality law will become more harmonised over time, and may fill in some of the gaps within national systems,^71^ any rights-based assessment tool such as a Human Rights Impact Assessment (HRIA) will still require adaptation to local contexts, reflecting the particular scope and requirements of equality law in the jurisdiction concerned. The contribution of this paper is to consider equality law as a supplement to a DPIA, with specific reference to the UK. Here, the domestic implementation of both the ECHR and (historically) EU equality law has produced the additional safeguard of the public sector equality duty, which illustrates the concrete added value equality law can bring when integrated alongside data protection frameworks.

### 4.1 The PSED

The Public Sector Equality Duty was introduced in the Equality Act 2010. It contains some of the lineage of previous non-discrimination duties in English law. Like the GDPR’s DPIA obligation, the PSED is essentially a reflective requirement: a necessary pause before an action is taken, to ensure appropriate evidence of its lawfulness. Before the relevant action is taken, section 149 of the Equality Act 2010 requires public authorities to have ‘due regard’ to their duty to:

(a) *eliminate discrimination, harassment, victimisation and any other conduct that is prohibited by or under this Act;*
(b) *advance equality of opportunity between persons who share a relevant protected characteristic and persons who do not share it;*
(c) *foster good relations between persons who share a relevant protected characteristic and persons who do not share it.*

The duty applies when a public authority ‘exercises its functions.’ In practice, this covers most of the authority’s outward-facing activity concerning the public, as opposed to its internal functions (e.g. as an employer). When a public authority processes a large volume of personal data for analytical purposes, this will almost certainly be in the exercise of its functions. As such, it is safe to assume that the use of Big Data and AI in the public sector will be subject to the PSED.

### 4.2 Bridges v South Wales Police

To illustrate the DPIA-PSED alignment in practice, it is helpful to look outside the healthcare sector, to a case where both obligations have been challenged within the Courts. In this example, South Wales Police (also a public authority) were judicially reviewed for their failure to conduct an adequate DPIA, and for breaching the PSED. A review of this case suggests that these two legal breaches arose, in fact, from the same omission: a failure to systematically review the risks of the software they used on the general public.

A key strength of the DPIA is its capacity to prompt reflection when novel technologies are deployed by public authorities. An inadequate DPIA thus represents a missed opportunity to review the evidence available, before data processing begins. The High Court judgment in *Bridges v South Wales Police* was suggested to be the first time any court in the world had considered the use of Automated Facial Recognition (‘AFR’) Software.^72^ The claim for judicial review of South Wales Police’s use of AFR was brought by Edward Bridges, with the support of the human-rights non-governmental organisation Liberty.

The subsequent judgment of the Court of Appeal in *R (Edward Bridges) v The Chief Constable of South Wales Police*^73^ contains careful analysis of DPIAs within public authority decision-making. The two grounds of appeal which are relevant for this paper are set out at paragraph 53, and can be summarised as follows:

• South Wales Police breached the Data Protection Act 2018 (which imports the GDPR) by failing to conduct an adequate DPIA;
• In particular, the DPIA was inadequate because it contained an error of law, in assuming that the right to privacy under Article 8 ECHR did not apply.
• South Wales Police also breached the PSED by conducting an inadequate Equality Impact Assessment, which did not sufficiently address the risks of indirect discrimination.^74^

These grounds were successful: the Court of Appeal held that the DPIA was insufficient, and that the risk of indirect discrimination was insufficiently recognised by South Wales Police. In short, the Court of Appeal concluded that insufficient efforts had been made by the police to investigate the potential for racial or gender bias in the software they had licenced,^75^ leading to breaches of both data protection law and the public sector equality duty. This case thus illustrates the natural alignment of these obligations: had South Wales Police conducted a more thorough and legally accurate DPIA, they would have stood a better chance of identifying the risks of indirect discrimination on racial and gender grounds, and thus of complying with the Public Sector Equality Duty. This ‘more thorough’ DPIA could, I suggest, have been completed by aligning the DPIA and Equality Impact Assessment (‘EIA’) processes, to create a UK version of a ‘DPIA+’ model. NHS England has adapted the generic ‘EIA’ template, used in the UK public sector, to capture risks of health inequalities, as well as discrimination. As such, the resulting ‘Equality and Health Inequalities Impact Assessment’^76^ is an existing process that could readily be incorporated into a DPIA review of a programme of data processing. At its core, the ‘EIA’ model sets a precedent for breaking down risks of harm systematically, according to groups with particular protected characteristics, evaluating this each risk and identifying strategies of mitigation. To simplify NHS England’s template EHIA,^77^ this looks roughly as follows Table 1:

**Table 1:** Simplified EHIA template, which could be incorporated into an evaluation of harm under a DPIA.

Protected characteristic groups	Summary explanation of the main potential positive or adverse impact of your proposal	Main recommendation from your proposal to reduce any key identified adverse impact or to increase the identified positive impact
Age: older people; middle years; early years; children and young people.	Preventing cases of Covid-19 in older members of the population vs risk of minimising the prevalence in this population via under-reporting.	
Disability: physical, sensory and learning impairment; mental health condition; long-term conditions.	Preventing cases of Covid-19 in people with serious health conditions vs risk of misrepresenting the prevalence in this population via under-reporting.	

### 4.3 DPIA+ EIA = DPIA+

The advantages of combining the DPIA and PSED evaluation processes are potentially manifold, including the more ineffable benefit of drawing from two evolving schools of thought, which can both help refine broad concepts such as ‘fairness.’^78^ Prosaically, there is also the practical advantage of avoiding duplication of time and effort, where there is overlap. The two advantages considered in this subsection are 1) drawing together an expanded pool of stakeholders, by combining characteristics protected under the GDPR and the Equality Act and 2) mutually reinforcing each other’s requirement to investigate risk of discrimination. To better align with the stated advantages of a ‘DPIA+’ in the UK, the following subsections clarify the PSED’s scope, and the statutory duty to investigate risk of discrimination.

#### 4.3.1 Expanded scope of evaluation

The PSED directs attention to the risk of discrimination on the basis of ‘protected characteristics,’ which overlap with the GDPR’s special categories of data without mirroring them entirely:

The Table 2 above suggests the areas of overlap. The GDPR is— perhaps— more aimed at novel forms of informatics, such as genetic and biometric data, than the demographic data that the Equality Act 2010 identifies, nonetheless, as potential loci of discrimination. The combined effect of the two amounts to a broader span of characteristics, which may still be helpful to consider together— for example, under a combined consultation process, to help identify groups at greater risk of harm. To use *Bridges* as an example, the identified risk of discrimination was on the basis of race and gender, and the latter could have been excluded from a DPIA for falling outside the scope of the GDPR’s ‘special categories’ of personal data.

**Table 2:** Protected characteristics under equality and data protection laws.

Equality Act 2010, s.149(7)	GDPR, Article 9(1)
Age	
Gender reassignment	
Sex	
Disability	[Data concerning health]
Pregnancy and maternity	[Data concerning health]
Race	Race or ethnic origin*
Religion and belief	Political opinions Religious or philosophical belief
Sexual orientation	Sex life or sexual orientation
	Trade union membership
	Genetic data
	Biometric identifiers

In other jurisdictions, the local application of equality law may have a different effect. For example, there could be a different contribution in countries that have ratified Protocol 12 of the ECHR^79^ (which established a broader scope of antidiscrimination law, beyond Convention rights). The impact of equality law on a DPIA in other European jurisdictions merits further study, but is outside the scope of this article. Likewise, equality law does not apply uniquely to the public sector, and could supplement a DPIA within the context of (e.g.) employment within the private sector. However, this article focuses on the Public Sector Equality Duty, which only applies to public authorities in the UK. This is partly because of the motivating concern of this study: the use of Big Data by public authorities to make automated decisions about citizens (see section 2). But also because the ‘PSED’ imports the particular benefit of the Equality Impact Assessment as a template for evaluation, which is worth considering as a supplement to structure the harms identified in a DPIA according to protected characteristics.

A further limitation is that equality-inflected DPIA cannot capture harms arising from groupings based on characteristics which are not legally protected. For example, this is not a solution for the opaque ‘algorithmic groups’ Wachter discusses (e.g., eye colour, zodiac sign, or other arbitrary criteria).^80^ But the case study here deliberately targets characteristics empirically linked to health outcomes during Covid-19 outcomes—age, disability, and race/ethnicity—which better align with Equality Act protected characteristics and (in part) the GDPR’s special categories. In life-and-death public-health settings, this overlap is precisely where equality law adds operational value to prospective DPIA.

#### 4.3.2 Duty to Investigate

Another contribution of s.149 EA 2010 is the duty to investigate risks of discrimination. In addition to the statutory language of s.149 EA 2010, the PSED’s requirements have been developed in judicial commentary now approved by the Supreme Court^81^ in *R(Bracking) v Secretary of State for Work and Pensions*.^82^ The court elaborated that the duty is proactive, substantive and may require active investigation:

The PSED must be fulfilled before and at the time when a particular policy is being considered. […]The duty must be exercised in substance, with rigour, and with an open mind. It is not a question of ticking boxes. If the relevant material is not available, there will be a duty to acquire it and this will frequently mean that some further consultation with appropriate groups is *required.*^83^

The DPIA also has, to some extent, a consultation requirement in the form of Article 35(9):

*Where appropriate, the controller shall seek the views of data subjects or their representatives on the intended processing, without prejudice to the protection of commercial or public interests or the security of processing operations.*

The crucial words, here, are ‘*where appropriate*.’ There are many circumstances where the data controller has discretion to decide it is not ‘appropriate’ to consult with data subjects. In the case of public authorities, it is all too easy for public interest or security to be cited as reasons militating against transparency. In the context of the PSED, however, public authorities seem to have less leeway. In the words of the Court of Appeal judges in their combined *Bridges* judgment:

*If the relevant material is not available, there will be a duty to acquire it and this will****frequently****mean that some further consultation with appropriate groups is required* (emphasis added).^84^

Where a DPIA is conducted alongside consideration of the PSED, therefore, the GDPR’s ‘*where appropriate*’ caveat should be treated with more caution. These investigatory efforts do not end with what the ICO term ‘participatory design’ in their guidance on the use of AI,^85^ and can also require expert assessment. In the case of *Bridges*, the Court of Appeal carefully reviewed the technical evidence on the risk of bias in AFR. While they did not make any finding that the specific software procured by South Wales Police posed a risk of bias, they noted the general risk of disproportionate error affecting Black people and women in AFR software. As such, the lack of investigation, and evidence that their software did *not* contain discriminatory bias, was sufficient to constitute a failure of the PSED.^86^

South Wales police would have had a reasonable degree of discretion as to the nature of this investigation, but one possibility would be a proxy assessment. A proxy analysis is one of the tools the ICO recommends as part of its AI guidance^87^— a test to see whether an algorithm falsely correlates a data feature (such as appearance on a watch list) with a protected characteristic (such as race or gender). Based on the Court’s findings in *Bridges*, proxy assessment prior to the deployment (or even procurement) of new software by a public authority could have been a useful tool to investigate risk of discrimination under the DPIA and the PSED. Combining the two is, at least, an efficiency, but also an opportunity to draw from the guidance available under both statutes, and gain a fuller account of discrimination risk. As the Court of Appeal noted in their combined judgment:

[181] *We acknowledge that what is required by the PSED is dependent on the context and does not require the impossible. It requires the taking of reasonable steps to make enquiries about what may not yet be known to a public authority about the potential impact of a proposed decision or policy on people with the relevant characteristics, in particular for present purposes race and sex.*

The combined effect of DPIA and the PSED thus serves to mutually reinforce their requirements to proactively investigate the risk of bias, prior to an action being taken. Had South Wales Police considered the data protection and equality act duties holistically, these should have prompted investigation of the risks of indirect discrimination, which could in turn have prevented the omissions which the Court of Appeal ultimately found to be breaches of the PSED.

In short, the combination of the DPIA and the PSED is promising both in its resulting expansion of legal scope (the greater range of personal characteristics to be considered) and also in the mutual reinforcement of the respective investigative requirements. This combination would have been useful as a proactive consideration in the South Wales Police case discussed above, but this is not the only example of its utility.

To explore the potential benefit of combining equality and data protection law considerations, the next section returns to the main case study of this paper: the NHS Covid-19 Data Store.

## 5 The DPIA for the Covid-19 data store

### 5.1 The beginning

The UK government announced the construction of the NHS Covid-19 Data Store in March 2020. A large part of the store was built, and put into operation, before an accompanying Data Protection Impact Assessment was published. 88 The Secretary of State’s instructions to share data were first issued in March 2020, with the accompanying DPIA published in June 2020.^89^ We do not know when the DPIA process for the Data Store was initiated, or what form it took. Nevertheless, healthcare providers were legally obliged to disclose patient information for a broad array of ‘Covid-19 purposes,’ before it was publicly established that the intended Covid-19 Data Store was compliant with data protection law.

As an initial point, which is crucial for this paper—as much impact assessment as possible should be conducted, and published— prospectively. Article 35(1) GDPR is clear that a DPIA should take place ‘*prior to the processing.*’ Even in an urgent situation, therefore, a DPIA should be conducted and published at least in an initial form before the processing is legally mandated by central government. This means that health and social care providers, as well as the patient population, are given some assurance that the legally mandated disclosure of information has been reviewed from a data protection perspective, and at least a provisional analysis of harm has been conducted. This should not require any delay, but instead provide a complementary evaluative process, to ensure the limitations of the data can be understood and mitigated as the resource is built. In this sense, lawyers and data scientists have a joint interest in data quality, with early evaluation meeting the concerns of both.

### 5.2 The ‘COPI’ notice

The legal origin of the Data Store was an instruction from the Secretary of State for Health and Social Care, telling healthcare providers to share patient information for Covid-19 purposes. This instruction took the form of a ‘COPI notice.’ ‘COPI’ notices are made under the Health Service (Control of Patient Information) Regulations 2002. These notices can appear to provide a definitive ‘legal basis’ for subsequent data processing—i.e., complete assurance that all processing is lawful. For example, the letter to NHS Digital states:

‘*I consider this Notice is necessary so that NHS Digital can lawfully and efficiently disseminate confidential patient information’*^90^

This is true to the extent that a COPI notice provides a starting point for lawfulness. It ensures that the use of patient data is lawful, at least according to some of the applicable law. It gives healthcare providers a basis in law for the disclosure of patient data, meaning it should not breach their duty of confidence. It should also satisfy GDPR Article 6(1)(e), which requires public authorities to have a foundation in law when they process personal data. It does not, however, ensure that the processing will comply with other areas of law, such as the Public Sector Equality Duty under s.149 Equality Act. It is also no substitute for a DPIA. If a subsequent DPIA identified a high risk of harm to data subjects, which could not be sufficiently mitigated, the processing would still breach the GDPR, and thus be unlawful.^91^

### 5.3 The Data Store DPIA

The responsibility for conducting a DPIA for the Data Store fell to the assumed sole data controller for the NHS Data Store: NHS England.^92^ There is an inherent tension in the application of the GDPR, here, as the Regulation envisages that the controller will have a significant amount of say in the means and purposes of processing—either alone or ‘*jointly with others.*’^93^ This assumption is less straightforward when the main architecture of the processing has already been determined by the Secretary of State through a COPI notice. This question should have been considered within the DPIA itself, as a foundational point of accountability (e.g., whether the government department which mandated the data processing is, in fact, a joint controller).

Otherwise, the DPIA for the Covid-19 Data Store published by NHS England —now updated to version 5.a^94^— provides a thorough, factual picture of the processing operations involved in the construction and operation of the Data Store. In doing so, it apparently succeeds in providing a systematic review of the intended processing; the first requirement of a DPIA under Article 35(7)(a) GDPR. Some consideration is also given to the necessity of the processing, per Article 35(7)(b), with the document citing the need to centralise information for the government, and the superior quality of national records compared to regional ones.^95^

The element of the DPIA which is the focus of this paper— investigation of risks to data subjects—is less apparent. There are, essentially, four requirements of a DPIA under the GDPR:

a) *a systematic description of the envisaged processing operations and the purposes of the processing[…]*^96^
b) *an assessment of the necessity and proportionality of the processing operations in relation to the purposes;*
c) *an assessment of the risks to the rights and freedoms of data subjects referred to in paragraph 1; and*
d) *the measures envisaged to address the risks, including safeguards, security measures and mechanisms to ensure the protection of personal data and to demonstrate compliance with this Regulation taking into account the rights and legitimate interests of data subjects and other persons concerned.*

Satisfaction of a DPIA’s second two requirements c) and d) is not evidenced by NHS England’s published DPIA. Under ‘assessment of risk to data subjects,’ the document simply states *‘(t)he risk assessment contains security information which will not be published*.’^97^ This statement may not tell the full story, as NHS England has published a separate risk assessment within an Excel spreadsheet.^98^ But, as a starting point it is concerning. By excluding the detail of the risk assessment, it omits the most important evaluative exercise of a DPIA, and does not feed an assessment of risk into the overall narrative of its deliberation. Assessment of risks to the rights and freedoms of individuals is a compulsory part of a DPIA under the GDPR,^99^ and without it any account of ‘*measures envisaged to address the risks’*^100^ does not make sense. By failing to meet two out of the four statutory requirements for a DPIA, the published version falls significantly short of evidencing the evaluative exercise required by the GDPR.

The risk assessment Excel spreadsheet, published separately, improves on the blank redaction within the DPIA.^101^ Its content is appropriate, and identifies information security risks which will inevitably accompany the centralisation of NHS data. The risks to individuals listed in the spreadsheet are strongly inclined towards information security, however, and are not linked in any detail to the particular purpose of the Covid-19 Data Store, for example:

*The context in which information is used or disclosed can change over time, leading to it being used for different purposes without people’s knowledge.*

This is probably true, but a little generic. The words ‘Covid-19’ do not feature at all in the risks identified in this spreadsheet, nor any reference to the risk of skewed analytics impacting service provision, nor whether this is a heightened risk in respect of demographics vulnerable to under-representation within NHS datasets. These risks are explained further in the following subsection, with the benefit of subsequent studies. Although these studies were conducted retrospectively, that does not mean the risks of discrepancy they investigated were impossible to anticipate before the Data Store was built. The Equality Act 2010, and the General Data Protection Regulation, set out a range of characteristics which are (respectively) protected, or special categories of information. As the GDPR itself states, they are special characteristics because they are recognised as potential loci of (indirect^102^) discrimination, or other threats to fundamental rights and freedoms.^103^

These risks were possible to anticipate in March 2020, and could not have been iteratively reviewed as the Store was constructed and operated. A (non-exhaustive) list of considerations this could have included is detailed below.

## 6 Risks of bias in the Covid-19 data store

While there are no certainties as to whether the following factors made a difference in the Data Store’s analytics, I suggest they would have merited consideration under a DPIA— particularly one reinforced by the Equality Act 2010. Under the template structure of an Equality Impact Assessment, risks of harm to different groups would be taken each in turn, according to their protected characteristics. To illustrate the added value of such an ‘EIA’ in identifying risks prospectively, this section takes a similar approach, and addresses distinct risks of data bias across four subsections.

### 6.1 Opt-outs

A constant source of potential bias in NHS data comes from the implementation of patients’ right to opt out from secondary uses of their health data. This can be a cause of concern for any policymakers using patient data to inform their decision-making. It is possible that some patient groups may opt out of secondary uses of their data at a higher rate, meaning they are under-represented in the final dataset available for analysis. For example, an Equality Impact Assessment for a regional NHS Secure Data Environment programme devotes a significant proportion of its analysis to the impact of the national data opt-out.^104^

Any data shared under a COPI notice from the Secretary of State should have been subject to the national opt-out.^105^ From the subsequent round table report, it seems the question of ‘*how to adhere to people opting out of their data being used by the health service’*^106^ was one of the initial delaying factors in the construction of the store.

As well as implementing the opt-out, it would have been important to consider its potential impact on the datasets. This is not necessarily a straightforward exercise. As Tazare and colleagues point out, one of the key limitations created by the opt-out is that it prevents the use of data for research— even research on the impact of the opt-out itself. This means there is limited demographic information about the individuals who are missing from NHS research data.^107^ Whether this data under-represents, or over-represents, certain demographics is thus difficult to establish. For example, the authors suggest that in 2013, opt-outs were higher among Black people than among White and Asian groups, but they were unable to complete a more up-to date analysis as ethnicity data were not available.^108^ They do also note, in relation to one database:

*Studies based on Clinical Practice Research (CPRD) databases, which consist of de-identified data from a large proportion of UK practices and do not include people who have opted out, suggest that the database was broadly representative of the UK population by age, sex, area-based deprivation, and ethnicity in May 2021 and before. This suggests that the low proportions of opt-outs at this time, and small differences between demographic groups, had minimal impact on overall representativeness at this stage.*

These findings are encouraging, but they seem only to relate to one patient database (the CPRD). The DPIA for the Covid-19 Data Store, on the other hand, lists 35 sources of information,^109^ which may vary in their representativeness. It bears repetition that the stated aim for the Data Store was to create ‘*a single source of truth*.’^110^ Those making decisions based on analytics within the store should have been warned about any gaps in their single source of truth. This is the kind of mitigating safeguard against indirect discrimination a DPIA could appropriately discuss: whether decision-makers should be given these caveats before allocating resources/ lifting restrictions based on these analytics.

### 6.2 Age

A major demographic at risk of indirect discrimination was made up of patients over the age of 80. Age was by far the greatest factor in the disparity of outcome of Covid-19, with patients over 80 being seventy times more likely to die following a positive test result than those under 40.^111^ Clearly, decisions about the response to Covid-19 would have a particularly significant impact on older patients as compared to people more likely to survive the virus.

In making decisions such as the placing or lifting of lockdown restrictions, or whether to focus clinical resources on care homes, the disproportionate impact of the decision on older people would have required consideration under the PSED. To do so properly, the risk needed to be represented as accurately as possible from the information available. If there was any doubt as to the representativeness of the data in the Data Store’s dashboards about patients aged 80+ (for example), these should be flagged.

Public Health England’s report (published in August 2020)^112^ suggests that deaths from Covid-19 in care homes were under-reported in March-May 2020. This is the kind of informational gap which could have under-represented the scale of the problem for this patient group within the Data Store dashboards, and where careful margins of error would be a useful safeguard, before decisions are taken. A simplified illustration would be:

**Table:** 

Risk	Harm	Mitigation
It is not certain that we have full, up-to date reports of the number of deaths from Covid-19 within care homes.	Care home residents are disproportionately older and disabled, and deaths within these groups may therefore be inadvertently minimised when resource allocation is considered.	Data limitations will be investigated and reviewed on an ongoing basis, and subject to external peer review. Data Store dashboards to flag the current margin of error, which will be reviewed and updated as often as possible.

This is not to say that the ultimate policy outcomes would, or should, have been different. Decisions with disproportionate impact on older patients could still have been taken, and justified under the Equality Act 2010.^113^ But the role of a DPIA is to prevent avoidable harm from data processing—such as harms stemming from unfair or inaccurate use of data. As such, the risk of older patients being under-represented within a decision-making resource should have been considered within an impact assessment.

### 6.3 Race

Race is another protected characteristic under the Equality Act 2010 which raises questions of representation within NHS data. Access to NHS services—and thus representation within NHS data—is a complex issue, driven by a number of factors. In the context of virtual patient cohorts, for example, the NHS itself suggests that ‘*insufficient ethnicity data capture, language and cultural barriers, and a misalignment between referral demographics and geographic profiles’* could be a reason for BAME under-representation.^114^

Likewise, if people from minority ethnic groups were less likely to access NHS care during the Covid-19 pandemic,^115^ this too could have distorted the government’s ‘*single source of truth’* about the prevalence of the disease in England. This is not a straightforward risk to assess—if anything, one London study suggested that minority ethnic patients were over-represented in hospital data, due to higher rates of hospitalisation.^116^ But in 2020, Public Health England reported higher rates of deaths for people in England who were born outside Europe,^117^ also citing potential barriers in accessing services.^118^ If these patients were less likely to access services, they may in turn have been less likely to feature in the Big healthcare Data informing the datastore. Racial minorities could therefore constitute another patient group (or set of groups) whose representation within the dashboards merited scrutiny, so that their needs were not inadvertently downplayed.

### 6.4 Disability

The risk of under-representation of care home residents has already been discussed. Another group of disabled patients who could also have been considered, however, are those with a learning disability.

This group was not among those highlighted by Public Health England in their August 2020 report on outcomes, but subsequent studies showed an association between learning disability and a higher risk of death from Covid-19.^119^ This raises questions of how well patients with a learning disability would be flagged within the government’s analytics. Williamson and colleagues point out that only an estimated 23% of people with learning disabilities are included on the national learning disability register, meaning that the association between the disability and mortality could well be higher than reported.^120^ This, they argue, is concerning for the evidence base informing decisions around vaccine prioritisation and other preventative measures for this group.

As above, this underlines the importance of iterative impact assessment that keenly targets gaps in any personal data used to inform government decision-making. It may not be realistic to expect NHS England to identify every patient group at higher risk of Covid-19 at the outset of a pandemic, but the public sector equality duty requires reasonable investigation of the risks of discrimination^121^—an investigation which should be iterative, and reviewed as fresh information comes to light.

### 6.5 The (Retrospective) DPIA+ for Covid-19

To reiterate: the above is not a criticism of any decisions taken in reliance on the Covid-19 Data Store, or a suggestion that any patient group suffered unlawful or unjustified impact from these decisions. The importance of the Data Store resource to inform decision-making is clear, and it is evident that a huge amount of work went into its urgent construction.^122^ However, combining DPIA with the public-sector equality duty in relation to health data governance— i.e. the DPIA+ model for which this paper advocates— would have significantly reduced the risks of biases. This is not a call for an overhaul of existing processes, but rather for careful refinement. Evaluation of data bias, and discrimination risks within a DPIA process could be a complementary, supportive process that helps improve the quality of the information resource as it is designed and built. There is emerging recognition of how data accuracy as a data protection principle can help ensure fairness in algorithmic processing.^123^ The evaluative model proposed here forms part of this alignment between technical accuracy and legal fairness.

## 7 Conclusion

This article has illustrated the added value of equality law in expanding a DPIA within the UK public sector. I have used the open-ended term ‘DPIA+’, as an acknowledgement of the range of laws that could supplement a DPIA in any given national context. But within the UK, bringing in approaches from equality law introduces a ready-adopted model to evaluate harms on the basis of protected characteristics, as well as a fact-sensitive duty to acquire evidence on the risks of discrimination, and engage potentially affected groups.

The reflection on the Covid-19 Data Store illustrates how this could work in practice. *Bridges v South Wales Police* illustrates that failures to scrutinise discrimination risk can simultaneously undermine DPIA adequacy and breach the PSED. Treating the DPIA and EIA/EHIA as a single, structured evaluation could have prompted earlier enquiry into data bias relating to age, race and disability. In life-and-death contexts like Covid-19, the case study of the NHS Covid-19 Data Store underscores why prospective, iterative assessment of bias, representativeness and downstream effects (e.g., care-home under-reporting, opt-outs, learning-disability under-registration) should be identified early. The goal is not to impede urgent action, but to ensure accurate, fair and explainable decisions where the potential for disparities across patient groups is most acute.

The proposal complements— rather than duplicates— the high-level HRIA model proposed by other authors,^124^ by adapting it within a binding national equality framework. The risks of Big Data processing in the public sector are not set to abate. Even if a failure comparable to the Dutch welfare benefit scandal can be avoided, controversies about how these data resources are built— and legitimated in law— continue. The Covid-19 Data Store discussed in this paper has now been migrated into a longer-term NHS Federated Data Platform. From the reported commentary, some confusion appears to persist as to whether this platform requires a fresh COPI notice to ensure its lawfulness, or can be built using general statutory powers.^125^ A DPIA has been published for this platform, but this document does not identify any of the risks of demographic-specific data bias which have been discussed in this paper.^126^ Clearly, large-scale processing of citizens’ data is set to remain a feature of public sector decision-making, and the DPIAs could be expanded to capture the full extent of the attendant risks. The value of equality law established, here, support further research into its contribution for DPIAs in other European jurisdictions.

During the preparation of this work the author used ChatGPT 5 to proof-read the final draft for typos and minor errors. After using this tool/service, the author reviewed and edited the content as needed and takes full responsibility for the content of the published article.

## Declaration of competing interest

None

## Data Availability

No data was used for the research described in the article.

## Footnotes

74 Unlike a DPIA, which the GDPR makes mandatory in some circumstances, an Equality Impact Assessment is *not* a mandatory assessment under the Equality Act 2010, but it is a recommended means of ensuring compliance with the PSED. See note 37.

96 The GDPR’s reference to ‘legitimate interests’ has been omitted here, as under Article 6(1)(f) GDPR public authorities cannot rely on this ground, and it is therefore out of scope for this paper.

## Footnotes

1 ‘Big Data’ is often characterised according to volume, variety, and velocity—some authors have also added ‘value’ and ‘variability’. There is a broad agreement that the data generated by healthcare services (the case study for this paper) contains these features, see e.g. Blagoj Ristevski and Ming Chen, ‘Big Data Analytics in Medicine and Healthcare’ (2018) 15 Journal Integrative Bioinformatics 3; Pantea Keikhosroki (ed). *Big Data Analytics for Healthcare : Datasets, Techniques, Life Cycles, Management, and Applications* (2022) (London, Academic Press).

2 Julie E Cohen, ‘Turning Privacy Inside Out’ (2019) 20 Theoretical Inquiries in Law 1, 1-22.

3 Michael Froomkin, ‘Big Data: Destroyer of Informed Consent’ (2019) 18 Yale Journal of Health, Policy, Law and Ethics 3. See also notes 4-9 below.

4 Linnet Taylor, Luciano Floridi and Bart van der Sloot (eds), *Group Privacy****:****New Challenges of Data Technologies* (2017) (Cham, Springer).

5 Aisling McMahon, Alena Buyx, and Barbara Prainsack, ‘Big Data Governance Needs More Collective Responsibility: The Role of Harm Mitigation in the Governance of Data Use in Medicine and Beyond’ (2020) 28 Medical law review 1, 155–182; Megan Doerr and Sandra Meeder, ‘Big Health Data Research and Group Harm: The Scope of IRB Review’ (2022) 44 Ethics and Human Research 4, 34-38.

6 Sandra Wachter and Brent Mittelstadt, ‘A Right to Reasonable Inferences: Re-Thinking Data Protection Law in the Age of Big Data and AI’ (2019) 2 Columbia Business Law Review, 494-619.

7 Ada Lovelace Institute, ‘Algorithmic impact assessment: user guide’ (8 February 2022) available from: https://www.adalovelaceinstitute.org/resource/aia-user-guide/ (accessed 12 March 2025).

8 Sylvie Delacroix and Neil D Lawrence, ‘Bottom-up Data Trusts: Disturbing the ‘One Size Fits All’ Approach to Data Governance’ (2019) 9 International Data Privacy Law 4, 236–252.

9 Effy Vayena and Alessandro Blasimme, ‘Health Research with Big Data: Time for Systemic Oversight’ (2018) 46 The Journal of Law, Medicine & Ethics 1, 119-129.

10 Regulation (EU) 2016/679 of the European Parliament and of the Council of 27 April 2016 on the protection of natural persons with regard to the processing of personal data and on the free movement of such data (United Kingdom General Data Protection Regulation); hereafter cited as ‘the GDPR.’

11 Alessandro Mantelero, ‘AI and Big Data: A blueprint for a human rights, social and ethical impact assessment’ (2018) 34 Computer Law & Security Review 4, 754-772.

12 Alessadro Ortalda and Paul De Hert, ‘Artificial intelligence human rights impact assessment’ in Alberto Quintavalla and Jeroen Temperman (eds.), *Artificial intelligence and human rights* (2023) (Oxford, Oxford University Press).

13 GDPR, Article 35.

14 Mantelero, note 11, 770. Ortalda and De Hert, note 12, 550.

15 Mantelero, note 11, 756. Ortalda and De Hert, note 12, 545.

16 Samuele Bertaina, Ilaria Biganzoli, Rachele Desiante, et al, ‘Fundamental rights and artificial intelligence impact assessment: A new quantitative methodology in the upcoming era of AI Act’ (2025) 56 Computer Law & Security Review 106101.

17 See also Alessandro Ortalda, Stefano Leucci and Gabriele Rizzo, ‘Anticipating compliance. An exploration of foresight initiatives in data protection’ (2025) 59 Computer Law & Security Review 106182.

18 Equality and Human Rights Commission, ‘Technical guidance on the Public Sector Equality Duty: England’ (April 2023), available from:

19 Doug Pyper, ‘The Public Sector Equality Duty and Equality Impact Assessments’ (2020) available from: https://researchbriefings.files.parliament.uk/documents/SN06591/SN06591.pdf (accessed 25 September 2025).

20 NHS Sussex Health and Care, ‘Equality and Health Inequality Impact Assessments (EHIAs)’, available from: https://www.sussex.ics.nhs.uk/nhs-sussex/our-values/equality-diversity-and-inclusion/equality-and-health-inequality-impact-assessments-ehias/ (accessed 25 September 2025).

21 For example, NHS England and NHS Improvement, ‘Equality and Health Inequalities Assessment (EHIA) for the NHS app’ (March 2020), available from: https://www.whatdotheyknow.com/request/nhs_app_usage/response/2120382/attach/3/EHIA%20NHS%20App%20UPDATED%20January%202022%20Redacted.pdf (accessed 25 September 2025).

22 Note 11, 755.

23 Discrimination in relation to the right to private life, under Articles 8 and 14 ECHR.

24 European Parliament, Parliamentary question O-000028/2022, ‘The Dutch childcare benefit scandal, institutional racism and algorithms’ (28 June 2022) available from: https://www.europarl.europa.eu/doceo/document/O-9-2022-000028_EN.html (accessed 12 March 2025).

25 Jane Croft, ‘Post Office tried to ‘hush up’ case of worker who killed himself, inquiry hears’ (26 April 2024), available from: https://www.theguardian.com/uk-news/2024/apr/26/post-office-tried-to-hush-up-martin-griffiths-case-inquiry-hears (accessed 12 March 2025).

26 Melissa Heikkilä, ‘Dutch scandal serves as a warning for Europe over risks of using algorithms’ (29 March 2022) available from: https://www.politico.eu/article/dutch-scandal-serves-as-a-warning-for-europe-over-risks-of-using-algorithms/ (accessed 12 March 2025).

27 Robert Booth, ‘Revealed: bias found in AI system used to detect UK benefits fraud’ (6 December 2024) available from: https://www.theguardian.com/society/2024/dec/06/revealed-bias-found-in-ai-system-used-to-detect-uk-benefits (accessed 25 September 2025).

28 NHS England, ‘About the NHS AI Lab’ https://transform.england.nhs.uk/ai-lab/about-the-nhs-ai-lab/ (accessed 25 September 2025).

29 Department for Health and Social Care, ‘Data saves lives: reshaping health and social care with data’ (15 June 2022) available from: https://www.gov.uk/government/publications/data-saves-lives-reshaping-health-and-social-care-with-data/data-saves-lives-reshaping-health-and-social-care-with-data (accessed 12 March 2025).

30 The Public Law Project has obtained evidence of 55 automated systems used by the UK government, see ‘The Tracking Automated Government register’ (updated October 2023) available from: https://publiclawproject.org.uk/resources/the-tracking-automated-government-register/ (accessed 25 September 2025).

31 Information Commissioner’s Office, ‘NHS Blood and Transplant’ (3 March 2023) available from: https://ico.org.uk/action-weve-taken/enforcement/nhs-blood-and-transplant/ (accessed 12 March 2025).

32 Matthew Gould, Indra Joshi and Ming Tang, ‘The power of data in a pandemic’. (28 March 2020) available from: https://digileaders.com/the-power-of-data-in-a-pandemic/ (accessed 25 September 2025).

33 Public Health England, ‘Disparities in the risk and outcomes of COVID-19’ (August 2020): Disparities in the risk and outcomes of COVID-19 (publishing.service.gov.uk) (accessed 25 September 2025).

34 Elizabeth Williamson et al, ‘Risks of covid-19 hospital admission and death for people with learning disability: population based cohort study using the OpenSAFELY platform’ (2021) 374 BMJ 1592.

35 NHS England, ‘Data Protection Impact Assessment: NHS COVID-19 Data Store’ (16 February 2022), available from: https://www.england.nhs.uk/wp-content/uploads/2022/02/data-protection-impact-assessment-nhs-covid-datastore.pdf (accessed 25 September 2025).

36 See notes 20, 21 and 76.

37 Note 13.

38 Note 32, 6.

39 See the general definition of Big Data, note 1.

40 Note 28.

41 Often called ‘sensitive data’ in practice, this term under Article 9 GDPR covers health-related data, as well as information about sex life, sexual orientation, race or ethnicity, all of which are likely to be included (to some extent) in an individual’s healthcare record.

42 Article 36, GDPR.

43 Although some update has been achieved through the introduction of the AI Safety Institute. See Department for Science, Technology and Innovation ‘AI Opportunities Action Plan’ (13 January 2025) available from: https://www.gov.uk/government/publications/ai-opportunities-action-plan/ai-opportunities-action-plan (accessed 12 March 2025).

44 See notes 11 and 12.

45 Article 4(7), GDPR.

46 Articles 12-23 GDPR.

47 Note 8, 239.

48 Ibid.

49 Under the European Convention on Human Rights, and the European Union Charter of Fundamental Rights, as acknowledged in the preamble to the GDPR.

50 *Lloyd v Google* [2021] UKSC 50, [108-109]. This judgment was decided on the Data Protection Act 1998, which was based on the previous EU Data Protection Directive, but the GDPR has not significantly altered this fundamental balance of responsibility.

51 Article 5 GDPR.

52 ICO Guidance, ‘How do we ensure lawfulness in AI?’ (28 October 2024) available from: https://ico.org.uk/for-organisations/uk-gdpr-guidance-and-resources/artificial-intelligence/guidance-on-ai-and-data-protection/how-do-we-ensure-lawfulness-in-ai/ (accessed 12 March 2024).

53 McMahon et al, note 5.

54 Or, at least, the combined principle of lawfulness, fairness and transparency under Article 5 (1)(a).

55 Maria Tzanou, ‘Data protection as a fundamental right next to privacy? “Reconstructing” a not so new right’ (2013) 3 International Data Privacy Law 2, 88–99, 89.

56 Edward S Dove, ‘The EU General Data Protection Regulation: implications for international scientific research in the digital era’ (2019) 46 Journal of Law, Medicine & Ethics 4, 1013-1030.

57 Orla Lynskey, ‘Deconstructing data protection: the “added-value” of a right to data protection in the EU legal order’ (2014) 63 International and Comparative Law Quarterly 3, 569-597.

58 Juliane Kokott and Christoph Sobotta, ‘The distinction between privacy and data protection in the jurisprudence of the CJEU and the ECtHR’ (2013) 3 International Data Privacy Law 4, 222–228.

59 Articles 12-22 GDPR.

60 Articles 24-43 GDPR.

61 Articles 51-59 GDPR.

62 Article 5 GDPR.

63 Article 35.3(b) GDPR.

64 Recital 51 GDPR.

65 Reuben Binns, ‘Data protection impact assessments: a meta-regulatory approach’ (2017) 7 International Data Privacy Law 1, 22–35.

66 The ICO are, strictly speaking, a regulatory authority independent of the government, but they nonetheless fall within an expansive understanding of the state.

67 For example, when the expansion of the Ultra Low Emission Zone was judicially reviewed in *London Borough of Hillingdon & Ors, R (On the Application Of) v Mayor of London (Re ULEZ Expansion)* [2023] EWHC 1972 (Admin).

68 *R(HM) v Secretary of State for the Home Department* [2022] EWHC 695 (admin).

69 European Union Agency for Fundamental Rights and Council of Europe*, Handbook on European non-discrimination law* (Luxembourg: Publications Office of the European Union, 2018), available from: https://fra.europa.eu/en/publication/2018/handbook-european-non-discrimination-law-2018-edition (accessed 26 September 2025).

70 Christa Tobler, ‘Equality and Non-Discrimination under the ECHR and EU Law A Comparison Focusing on Discrimination against LGBTI Persons’ (2014) 74 ZaöRV, 521-561.

71 European network of legal experts in gender equality and non-discrimination, ‘A comparative analysis of non-discrimination law in Europe 2024’ (1 January 2024) available from: https://op.europa.eu/en/publication-detail/-/publication/459e3462-1041-11f0-b1a3-01aa75ed71a1/language-en (accessed 26 September 2025).

72 [2019] EWHC 2341 (Admin), [1].

73 Note 19.

75 Note 19, [201-202].

76 For example, NHS England ‘Equality and Health Inequalities Impact Assessment (EHIA)’ (11 October 2022) available from: https://www.england.nhs.uk/long-read/nhs-england-equality-and-health-inequalities-impact-assessment-ehia/ (accessed 26 September 2025).

77 Ibid.

78 In a different context, Gefenas et al argue that data protection should be considered in conjunction with research ethics on issues such as ‘public interest,’ as these separate aspects of compliance can become institutionally siloed. Eugenijus Gefenas, Jurate Lekstutiene, Vilma Lukaseviciene, et al, ‘Controversies between regulations of research ethics and protection of personal data: informed consent at a cross-road’ (2022) 25 Medicine Health Care and Philosophy 2, 23–30.

79 Council of Europe, Protocol No.12 to the Convention for the Protection of Human Rights and Fundamental Freedoms.

80 Sandra Wachter, ‘The theory of artificial immutability: protecting algorithmic groups under anti-discrimination law’ (2023) 97 Tulane Law Review 2, 149–204

81 *Hotak v Southwark LBC* [2015] UKSC 30, [2016] AC 811, at [73] (Lord Neuberger PSC).

82 [2013] EWCA Civ 1345

83 Note 19 [175].

84 Ibid.

85 Information Commissioner’s Office, ‘Guidance on AI and data protection’, ‘Annex A: Fairness’ available from: https://ico.org.uk/for-organisations/uk-gdpr-guidance-and-resources/artificial-intelligence/guidance-on-ai-and-data-protection/annex-a-fairness-in-the-ai-lifecycle/ (accessed 17 March 2025).

86 Note 19 [164-202].

87 Note 82.

88 Institute for Government, ‘NHS Covid-19 Data Store and NHS National Data Platform Summary of a private roundtable’ (2022) available from: https://www.instituteforgovernment.org.uk/sites/default/files/2023-02/nhs-covid-datastore.pdf (accessed 17 March 2025).

89 The DPIA was first published 5 June 2020, see NHS England ‘Data Protection Impact Assessment: NHS COVID-19 Data Store’ available from: https://www.england.nhs.uk/publication/data-protection-impact-assessment-nhs-covid-19-data-store/ (accessed 17 March 2025).

90 Letter from Secretary of State for Health and Social Care to Sarah Wilkinson, Chief Executive of NHS Digital, (17 March 2020) available from: https://digital.nhs.uk/coronavirus/coronavirus-covid-19-response-information-governance-hub/control-of-patient-information-copi-notice (accessed 17 March 2025).

91 And, under Article 36 GDPR, this would have to be resolved with the Supervisory Authority (in this case, the Information Commissioner’s Office).

92 The DPIA (note 86, page 7) states that NHS England is the sole controller ‘*for any data legally shared with them under the NHS Control of Patient Information (COPI) Notice issued by the Secretary of State*’ but this statement is peculiar given that the Secretary of State had already made an initial determination of the purposes of processing within the COPI notice.

93 Per the definition in Article 4(7).

94 Note 32.

95 Ibid, pages 1 and 16.

97 Note 32, page 18.

98 NHS England, ‘DPIA Risk Register’ available from: dpia-nhs-covid19-risk-assessment-for-data-store.xlsx (live.com) (accessed 17 March 2025).

99 Article 35.7 (c) GDPR.

100 Article 35.7 (d) GDPR.

101 Note 95.

102 I.e. when an apparently neutral intervention disproportionately affects people with certain protected characteristics—see Hugh Collins and Tarunabh Khaitan ‘Indirect Discrimination Law: Controversies and Critical Questions’ in Hugh Collins and Tarunabh Khaitan (eds) *Foundations of Indirect Discrimination Law* (2018) (Oxford, Hart Publishing).

103 Article 51 GDPR.

104 Dominic Rowney and Suprasad Gavhane, ‘North East and North Cumbria Secure Data Environment programme— Health data sharing: Equality Impact Assessment’ (February 2024) available from: https://northeastnorthcumbria.nhs.uk/media/adbevmrg/ppie-equality-impact-assessment.pdf (accessed 17 March 2025).

105 NHS England, ‘Understanding the national data opt-out’ (16 May 2023) available from: https://digital.nhs.uk/services/national-data-opt-out/understanding-the-national-data-opt-out (accessed 17 March 2025).

106 Note 85, page 12.

107 John Tazare, Alasdair D Henderson, Jessica Morley et al, ‘NHS national data opt-outs: trends and potential consequences for health data research’ (2024) 8 BJGP Open 3, 2024.0020.

108 Ibid.

109 Note 32, pages 4-5.

110 Note 13.

111 Note 14, page 10.

112 Note 14, page 57.

113 Age being one of the protected characteristics for the purposes of the Equality Act 2010.

114 Tammy Lovell, ‘Black and ethnic minority people under-represented in virtual wards’ (3 June 2024) available from: https://www.digitalhealth.net/2024/06/black-and-ethnic-minority-people-underrepresented-in-virtual-wards/ (accessed 17 March 2025).

115 For example, due to immigration status, or linguistic/cultural barriers—See Public Health England report, note 14, page 40.

116 Annastazia E. Learoyd, Jennifer Nicholas, Nicholas Hart, et al, ‘Revisiting ethnic discrepancies in COVID-19 hospitalized cohorts: a correction for collider bias’ (2023) 161 Journal of Clinical Epidemiology, 2023, Pages 94-103, https://doi.org/10.1016/j.jclinepi.2023.06.014.

117 Ibid page 12, page 17.

118 Ibid, page 40.

119 Note 15.

120 Ibid.

121 Per the *Bracking* judgment, see note 79.

122 Note 85.

123 Elisabetta Biasin, ‘Why accuracy needs further exploration in data protection’ Proceedings of the 1st International Conference on AI for People: Towards Sustainable AI, CAIP 2021 (20-24 November 2021) Bologna, Italy.

124 See notes 11 and 12.

125 Lindsay Clark, ‘Key aspects of Palantir's Federated Data Platform lack legal basis, lawyers tell NHS England’ (5 September 2024) available from: https://www.theregister.com/2024/09/05/fdp_lacks_legal_basis/ (accessed 17 March 2025).

126 NHS England, ‘Overarching data protection impact assessment (DPIA) for the Federated Data Platform (FDP)’ (14 November 2024) available from: https://www.england.nhs.uk/long-read/overarching-data-protection-impact-assessment-dpia-for-the-federated-data-platform-fdp/ (accessed 17 March 2025).

